# An assessment of food-security-related articles and lessons learnt from Zimbabwe: A critical analysis

**DOI:** 10.4102/jamba.v11i1.799

**Published:** 2019-07-23

**Authors:** Tapiwa Muzerengi, Ernest Khalema

**Affiliations:** 1 Department of Community Development, University of KwaZulu-Natal, Durban, South Africa

## Introduction

A number of scholars have written intensively and extensively about food security issues with regard to availability, accessibility, vulnerability, utilisation and stability across continents. However, indications are that, while there is a lot of literature on the food security phenomenon, many scholars have not been able to develop and prescribe food security frameworks unique and specific to a given area in relation to its geo-political, socio-economic setting and available resources, resulting in acute food shortages in many poor communities. This article analysed scholarly articles by other authors on food security and identified contributions and gaps that are going to be addressed by policy, and aims to contribute to new body of knowledge and practice.

### Ethical considerations

The permission to collect data was given by the Provincial Administrator for Matabeleland South Province.

## Critical and systematic analysis of food security articles

Gillo (2017) talks of food security in the context of climate change awareness and effects on food production. As indicated by the abstract and introduction of the article, the thrust of the research was to find out if farmers were aware of the effects of the climate change and how these impede food production and availability. The research, which uses both primary and secondary sources of data, also aims to establish the coping mechanisms that farmers are employing to curb the effects of climate change on farming and food production.

It can be asserted that Gillo (2017) largely managed to cover the objectives of his study as indicated in the body of his article. The article managed to establish the awareness rate of climate change in farmers aggregated by age, sex and level of education, and this is illustrated by means of tabulations, charts and statistical descriptions. The effects of climate change, which have led to decline in food production levels, have been brought out in the article. The coping strategies the farmers are employing to avert the effects of climate change have also been clearly brought out in the article and these include crop diversification including adoption of drought-resistant crops and small-scale irrigation schemes.

However, the article did not cover much ground on what needs to be done to address the problem of climate change on food production. The article ends by a paragraph of recommendations and conclusions without giving in-depth solutions to this topical issue, and it tries to cover this gap by means of developing policies that are context-specific and addresses cross-cutting issues affecting food in Africa.

Chitongo and Munyati (2017) analysed food security in the context of poverty eradication and livelihood outcomes before and after implementation of the irrigation scheme in the case study area. Issues of food availability and accessibility with connection to irrigation development have been discussed in this article. As is set out by the study, the objectives of the study have been well brought out in the abstract and well-developed in the body of the article. The research indicates an improvement in the food security situation and the general standard of living in terms of ability to pay hospital bills, ability to buy groceries and ability to pay school fees and asset ownership emanating from irrigation development. The research findings that were collected through focus group discussions, key informant interviews and questionnaires are presented in the form of narrative descriptions with the use of charts and graphs showing trends of the levels of production before and after inception of the irrigation scheme. From the observed trends, farmers have been able to realise better yields in terms of beans, wheat, maize and potato tonnage and this all feeds to an improved food security situation at household level in Chirumanzu District.

Chitongo and Munyati (2017) in their article further discuss about the challenges affecting the farmers to realise their full potential in terms of production such as pests and diseases, and lack of financial and human capital. However, the article lacks enough coverage on what really needs to be done for irrigation development in Chirumanzu. There is a mere mention of the need for government support and farmer capitalisation in the abstract, but there is no specific section covering recommendations needed for improvement of the Musena irrigation scheme in Chirumanzu in the body of the article. For food security interventions to yield measurable results, there is a need to develop specific policies unique to the given case study, and this is what this article tries to address in Africa.

Andersen (2009) in his article ‘Food Security: Definition and Measurement’ grapples with the definition of food security in the context of household food security and vulnerability indicators, estimation of food security and magnitude of food security. The article manages to articulate various definitions which have been attached to the concept of food security and goes on to suggest that it can be a useful measure of household and individual welfare, particularly if combined with estimates of household food acquisition and allocation behaviour. Andersen (2009) indicates that if nutritional security is the goal of interest, estimates of access to food should be combined with estimates of access to clean water and good sanitation to make the definition and measurement of food security complete.

While it can be acknowledged that the article wholesomely defines the subject matter, it is rather limited in citation of specific examples or case studies to support the discussion. There is no attempt by the author to zero down on a specific case study area in equating his definition of food security and its measurement. Thus, food security issues need to be discussed in an area-specific manner as in most instances each area or geography presents its own specific challenges which need specific interventions.

Gupta and Wright (2017) in their article, ‘Situational nutritional analysis of Idu Mishmi tribes of Arunachal Pradesh, north-east India’, bring out the issue of nutritional balance as an important aspect of food security. The article, which is exploratory in nature, employed structured interviews, focus group discussions and key informant interviews to gather data on dietary patterns and nutrition adequacy, cultural beliefs surrounding food and impact on nutritional health. In terms of its findings, the research managed to establish that the tribal people were consuming a two-meal pattern diet consisting of high carbohydrate, low fat content, which was poor in vitamin A, thiamin, riboflavin, niacin, B12, vitamin C, calcium and iron. Anthropometric analysis showed one-fourth of children aged 2–9 years were underweight and 7% were stunted. This means that deficiency in some nutrition values points to a situation of food insecurity even when there is abundance of a certain type of food.

In this regard, it can be asserted that the work by Gupta and Wright (2017) is essential in bringing out the various facets of food security with regard to nutrition and health of the people in communities as this is depicted in the article through graphical presentations, tables, charts as well as narratives. However, the article did not give much insight on what needs to be done to promote a balanced diet as an important aspect of food security. The authors only mentioned the importance of nutritional health education in their concluding paragraph without giving a detailed picture of a framework to follow in promoting nutritional balance as an important aspect of food security.

Nantale (2017) in ‘Prevalence and factors associated with food insecurity among women aged 18–49 years in Kampala Slums Uganda; A mixed methods study’ takes a gendered approach to food security issues. The study which used a mixed methods approach in gathering data made use of interviewer administered questionnaire, key informant interviews and focus group discussions to gather evidence. The study managed to serve its purpose and established a high prevalence of food insecurity among women aged 18–49 years in an urban slum of Kampala, thereby bringing out vulnerability issues. Factors leading to high prevalence of food insecurity among women in slums are well articulated in the body of the article and are presented in tabular representations, charts and descriptions. For example, the increased household expenditures such as having more than one school going child coupled with low earning were associated with food insecurity.

The article ends by making recommendations on what needs to be done to empower women living in slums to overcome the problem of food insecurity. It cites that in order to reduce the proportion of food insecurity and the effects of food insecurity on maternal child health, there is a need to set up economic empowerment programmes for women in slums. Other studies are needed to create sufficient evidence to influence urban policy makers so that appropriate interventions against food insecurity are initiated. Thus, the article brings about an interesting aspect of inclusivity in dealing with the problem of food security.

However, despite the article being spot on in pinpointing the prevalence of food insecurity, the factors influencing such prevalence and recommending the involvement of vulnerable women in solving the food security equation, there is a need to further develop frameworks or working platforms or action plans which can help to promote such inclusion of all vulnerable communities and individuals such as women and children.

Mbuthia et al. (2017) take a look at food security with reference to environmental changes and how these affect food security at the household level. In the article entitled ‘Environmental determinants to household food security in Kyangwithya West Location of Kitui County’ which made use of key informant interviews and focus group discussions in the extraction of data, the authors established that high temperatures, recurrent droughts and inadequate rainfall greatly affect food security levels at the household level. The situation becomes worse when the farmers are not able to take note of the weather changes timely. The study also managed to rank the weather determinants of food security with regard to severity and established that inadequate rainfall was the main cause of food insecurity in the study area. Variations are drawn using tables, data grouped into thematic areas and statistical narratives to paint a clear picture of the effects of environmental patterns on food security in the Kyangwithya, which is the case study area. Thus, environment is a key factor in determining food availability and as such food policy planners need to pay attention to environmental issues in dealing with food security problems.

Even though the authors managed to identify the environmental determinants of household food security in the case study area, recommendations as to how these can be dealt with are limited in the article. The article only points to the need for timely communication of weather forecast in a single statement as a remedy. The issue of weather changes is taking precedence in affecting food security and as such it is high time that authors wholesomely recommend ways and come up with frameworks and action plans to combat the effects of weather patterns on food security in communities.

Nakasone and Suvedi (2017) in ‘Small farmers and market economy: A case study of Dagomba in northern Ghana’ takes a look at how the economic system of a society affects food production of small farmers. The article traces the development of the agricultural sector from the government of Gerry Rawlings in the 1970s before the SAPs took centre stage in Ghana and how the structural adjustment programs (SAPs) and current policies have affected agriculture in small farmer holder communities. This is done by analysing trends in economic growth, farming systems and land tenure systems. The market economy development has seen the farming of cash crops like cocoa taking centre stage and this in a way has dealt a blow to the production of food crops. The market economy has also seen movement or migration of young productive individuals to cities in search of jobs thereby putting pressure on the remaining elderly to produce food for the families. Thus the type of the operating economic structure affects agricultural productivity in a greater way.

Nakasone and Suvedi (2017) through the use of household surveys collected between 2005 and 2017 managed to show the trends and changes which have been brought by the market economy in Ghana and how it has affected the food production. The authors indicate how an economic system can create disparities between a country’s regions as is the case in northern Ghana where cash crops cannot be produced and Southern Ghana where cash crops can be produced. Thus, the effects of the market economy on agricultural production is well articulated by the authors when they pointed out that the position of agriculture as a source of income in rural areas has declined rapidly in the past few years as the weight of agriculture as an income source has been rapidly decreasing. The research further forecasts the possibility of de-agrarianisation owing to the market economy in the northern Ghana where cash crops are not viable. The article ends by making recommendations for new production techniques and crop diversification in order to improve agricultural productivity.

In a nutshell, thus it can be viewed that food security and economic systems feed into each other and as such policy makers should take into consideration the disadvantages and advantages an economic system can bring to agricultural productivity in terms of food production. There is a need to develop food security frameworks which ensure that the food production does not suffer at the hands of prevailing economic systems.

Nyaguyo (2012) in a thesis entitled, ‘The impact of disasters on rural households’ livelihoods and food security situation: Case of Muzarabani Rural District’ is concerned with the effects of flood disasters on rural household livelihoods and food security in the Muzarabani district of Zimbabwe. The research highlights the history of disasters in Zimbabwe, how they affect communities before zeroing in on Muzarabani flood disasters and their impact on the community. Through the use of both qualitative and quantitative data techniques, the researcher manages to extract valuable information on the subject matter, that is, the impact of flood disasters on Muzarabani community with regard to livelihoods and food security. Information on the trends and changes on asset base in terms of livestock like chickens, goats and cattle is well articulated through use of graphs, tables and narrative descriptions of percentages and other statistics. The impact of the floods on the food reserves is well depicted and pictured in the research. The overall observation is that floods severely and negatively affect food security status of poor and female-headed households in Muzarabani owing to lack of timely responsive measures to eminent floods.

The research concludes by running through a list of recommendations for addressing flood disasters and their impact on the livelihoods and food security situation in terms of early warning systems, creation of a community food reserve and relocation of people to high ground level. However, while the research can be applauded for bringing out the issue of flood disasters as one of the major factors affecting rural livelihoods and their food security situation, there is, however, a need to take a step further to design strategies and frameworks to address the problem at hand. The framework should be specific to a given area and should be designed together with the people affected and should also aim to use available resources to make it sustainable and workable. The researcher points more to what the government should do instead of what the affected people themselves should do or initiate to curb the effects of flood disasters on them. In many instances, researchers have been able to bring out or unravel a problem, but they have not been able to prescribe specific solutions to the problem in terms of an action plan.

Muchadeyi (2013) in a thesis entitled, ‘Aid agencies and sustainable livelihoods in rural communities: Case of Zaka district’ grapples with the work of donor agencies and how they have affected rural livelihoods in the drought-prone areas in Zimbabwe. The food security situation in the Zaka district is unravelled by the researcher and how the donor agencies’ food relief programmes have come in handy to avert the dire health and life-threatening situation of the vulnerable community. In this regard, the work of development agencies in promoting accessibility of food, this helps vulnerable communities to break the vicious cycle of food insecurity.

The research objectives are covered in the introductory chapter. The research which was qualitative in nature employing focus group discussions, key informant interviews and self-administered questionnaires while acknowledging the noble work being done by donor agencies in vulnerable communities managed to establish that the development agencies have created a serious dependency syndrome in the communities as some have shunned working in their own fields even in better seasons banking on food handouts from donors. The research established that donor agencies’ programmes are not home grown as the respondents to the study questions indicated that they have not participated in designing of programmes that are brought by donors to them. This creates a serious and unsustainable food security situation, which would leave the beneficiaries of the programmes in poverty and acute food shortages upon donor exit.

Although the research was able to establish the negative impact of donor agencies’ food handouts on vulnerable food deficit areas – in this case the Zaka District of Masvingo Zimbabwe – limitations are that the researcher did not manage to prescribe a strategic action plan to address the problem in the Zaka district specifically. The recommendations made by the research are rather too general and do not take into cognisance the resources available in Zaka and how these resources can be specifically used to address the problem of livelihoods and food security in the area.

Mapuranga and Muzerengi (2017) in an article named, ‘Impact of small-scale irrigation schemes in addressing food shortages in semi-arid areas: A case of Ingwizi irrigation scheme in Mangwe district, Zimbabwe’ establish the effects of irrigation development in addressing food shortages in semi-arid regions of Zimbabwe. The research managed to serve its purpose to establish the extent to which Ingwizi irrigation scheme has managed to address the problem of food shortage in Mangwe district. Thus bringing to the front, there is always a connection between irrigation development and food security or not.

The researchers in a research which is exploratory and qualitative in nature used focus group discussions, key informant interviews and observation to gather evidence from respondents and this augers well for the subject matter under discussion. Key issues were well brought out in the discussion through the thematic approach in data analysis. The research established that there is increased food insecurity in Mangwe district despite the presence of the Ingwizi irrigation scheme. The challenges affecting the scheme are well articulated and these include lack of government support, poverty and increased food imports. Thus, the article is able to address its key objective, which is bringing out the impact and the challenges of the irrigation scheme on food security situation in Mangwe district.

However, the article, as it is largely qualitative, did not point out much to trends in food production in terms of statistical narratives. There is no supporting numerical data to support how agricultural production at household level from the respondents in Mangwe district has been faring before and after the establishment of irrigation scheme in order to make comparisons based on statistics or figures. Furthermore, the research as an impact study ends by making a set of recommendations in the concluding paragraph, key being government support and subsidisation, and while this is a welcome gesture, this in itself is not enough as the problem of food insecurity need to be approached with well-developed work plans unique and specific to the problem area. In this research, the study will develop a food security framework specifically for Southern Africa taking cognisance of the geography, population, culture, politics and social dispensation of the area.

## Conclusion

In conclusion, it can be asserted that various scholarly researches have been able to establish key factors contributing to food security or insecurity in various communities such as droughts and changing weather patterns and the role played by donor agencies in critical food situations. However, the bulk of the literature available from scholars besides running through a set of recommendations to the food security problem did not provide specific and unique solutions to a given problem area. Most of the recommendations made are rather general and may not apply to a given area or other areas because of various factors ranging from difference in the geographical nature of an area and resources available in an area, both human and non-human resources, as well as the economic and political dispensation of the day. It is the aim of this article that a framework is developed to address the food security challenges in Zimbabwe based on the available resources and the economic structure which supports it.
